# *Acinetobacter baumannii *invades epithelial cells and outer membrane protein A mediates interactions with epithelial cells

**DOI:** 10.1186/1471-2180-8-216

**Published:** 2008-12-10

**Authors:** Chul Hee Choi, Jun Sik Lee, Yoo Chul Lee, Tae In Park, Je Chul Lee

**Affiliations:** 1 Department of Microbiology, Kyungpook National University School of Medicine, Daegu, 700-422, Korea; 2 Department of Pathology, Kyungpook National University School of Medicine, Daegu, 700-422, Korea

## Abstract

**Background:**

*Acinetobacter baumannii *is a nosocomial pathogen of increasing importance, but the pathogenic mechanism of this microorganism has not been fully explored. This study investigated the potential of *A. baumannii *to invade epithelial cells and determined the role of *A. baumannii *outer membrane protein A (AbOmpA) in interactions with epithelial cells.

**Results:**

*A. baumannii *invaded epithelial cells by a zipper-like mechanism, which is associated with microfilament- and microtubule-dependent uptake mechanisms. Internalized bacteria were located in the membrane-bound vacuoles. Pretreatment of recombinant AbOmpA significantly inhibited the adherence to and invasion of *A. baumannii *in epithelial cells. Cell invasion of isogenic AbOmpA^- ^mutant significantly decreased as compared with wild-type bacteria. In a murine pneumonia model, wild-type bacteria exhibited a severe lung pathology and induced a high bacterial burden in blood, whereas AbOmpA^- ^mutant was rarely detected in blood.

**Conclusion:**

*A. baumannii *adheres to and invades epithelial cells. AbOmpA plays a major role in the interactions with epithelial cells. These findings contribute to the understanding of *A. baumannii *pathogenesis in the early stage of bacterial infection.

## Background

Genus *Acinetobacter *are important opportunistic pathogens in hospital-acquired infections [1-4]. They cause various types of human infections, including pneumonia, wound infections, urinary tract infections, bacteremia, and meningitis. Of the currently known 31 *Acinetobacter *species [1,2,5], *Acinetobacter baumannii *is the most prevalent in clinical specimens. Numerous outbreaks caused by *A. baumannii *have been reported, which are of great concern in clinical settings [6,7]. Despite convincing evidence linking *A. baumannii *with human infections, the study of its pathogenic mechanism is still in its elementary stage.

Adherence of bacteria to epithelial cells is an essential step towards colonization and infection [8]. Bacterial adherence to host cells is mediated by fimbria or membrane components. Furthermore, many pathogenic bacteria are capable of invading non-phagocytic cells and evolve to survive within the host cells. The cellular invasion of bacteria contributes to evasion of humoral immunity, persistence in the host, and penetration into deep tissues. Bacterial pathogens gain entry to non-phagocytic cells via two mechanisms; a zipper-like mechanism and a trigger mechanism, which were initially classified based on morphological differences [9,10]. The zipper-like mechanism (receptor-mediated entry) requires the direct interaction of bacterial ligands to the host's cell surface receptors and involves local cytoskeletal rearrangement at the invasion site. In contrast, the trigger mechanism is initiated by the injected bacterial effector proteins delivered by the type III secretion system. The effector proteins regulate cytoskeleton dynamics and induce dramatic cytoskeletal rearrangements such as membrane ruffles. The previous study demonstrated that adherence of *A. baumannii *to human bronchial epithelial cells was mediated by fimbrial-like structures and entrapment of bacteria by cellular protrusions [11]. However, the mechanisms underlying invasion of *A. baumannii *in epithelial cells have not been explored so far.

Outer membrane proteins (Omps) of Gram-negative bacteria are key players in bacterial pathogenesis. OmpA of *Escherichia coli*, OspC of *Borrelia burgdorferi*, and Opa and OpcA of *Neisseria meningitidis *facilitate adherence to and invasion of bacteria in host cells [12-15]. OmpA of *A. baumannii *(AbOmpA) is the most abundant surface protein with a molecular mass of 38 kDa and plays a role in permeability of small solutes. AbOmpA purified from *A. baumannii *ATCC 19606^T ^and recombinant AbOmpA (rAbOmpA) bind to the surface of host cells and are localized in both the mitochondria and nuclei, which induce the death of host cells [16,17]. Based on previous findings that AbOmpA bound to and entered host cells, we hypothesized that AbOmpA is responsible for adherence to and invasion of *A. baumannii *in epithelial cells during the colonization and early stage of bacterial infection. In the present study, we investigated the potential of *A. baumannii *to invade epithelial cells and studied the role of AbOmpA in interactions of *A. baumannii *with epithelial cells.

## Results

### *A. baumannii *invades epithelial cells dependently on both the bacterial strains and cell types

**Figure 1:**
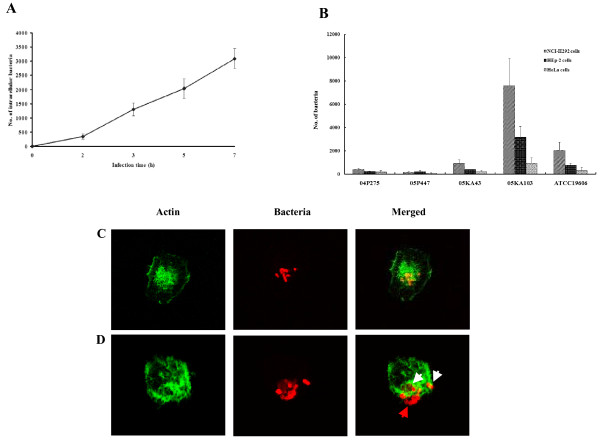
**Invasion of *A. baumannii *in epithelial cells**. (A) NCI-H292 cells were infected with *A. baumannii *ATCC 19606^T ^at an MOI of 100 up to 7 h. The colony-forming units were enumerated to measure the time-course of invasion. The result represents the mean ± standard deviation in duplicate wells and repeated a minimum of three separate times on separate days. (B) NCI-H292, HEp-2, and HeLa cells were infected with *A. baumannii *strains at an MOI of 100 for 5 h. (C) NCI-H292 cells were infected with *A. baumannii *05KA103 at an MOI of 100 for 5 h. Actin was stained with Alexa Fluor ^® ^488 phalloidin (green). Bacteria were stained with polyclonal anti-rabbit AbOmpA antibody, followed by a secondary antibody Alexa Fluor ^® ^568 (red). The analytical sectioning was performed from the top to the bottom of the cells. The figure represents a single section of the cells. (D). NCI-H292 cells were infected with *A. baumannii *ATCC 19606^T ^at an MOI of 100 for 5 h. Actin filaments have wrap-around-bacteria (white arrow). Red arrow indicates the extracellular bacteria. The figure represents all projection of sections in one picture.

To determine whether *A. baumannii *invaded epithelial cells, *A. baumannii *ATCC 19606^T ^and four gentamicin-susceptible *A. baumannii *isolates from clinical specimens were selected and gentamicin protection assay was performed. A multiplicity of infection (MOI) of 100 that was previously optimized in the adherence assay of *A. baumannii *was used in the cell invasion assay [11]. To optimize infection time, human bronchial NCI-H292 cells were infected for up to 7 h with *A. baumannii *ATCC 19606^T^. Intracellular bacterial counts increased steadily with incubation times (Fig. 1A), but cell death and destruction of cell monolayer appeared 5 h after infection. Therefore, we chose 5 h as the maximum infection period in the following study to exclude any secondary effect of cell death.

NCI-H292 cells were infected with four clinical *A. baumannii *isolates at an MOI of 100 for 5 h. Cell invasion of *A. baumannii *was different between bacterial strains: *A. baumannii *05KA103 was the most invasive (7,581 ± 2,365 cfu), whereas *A. baumannii *05P447 was the least invasive (151 ± 105 cfu) (Fig. 1B). To determine whether the cell invasion of *A. baumannii *is dependent on epithelial cell types, human bronchial NCI-H292 cells, human laryngeal HEp-2 cells, and human cervical HeLa cells were infected with *A. baumannii *strains. NCI-H292 and HEp-2 cells derived from the respiratory tract were more susceptible to cell invasion of *A. baumannii *than non-respiratory tract-derived HeLa cells (Fig. 1B). Confocal microscopic images provided direct evidence of intracellular localization of *A. baumannii *(Fig. 1C and 1D).

### *A. baumannii *invades epithelial cells by a zipper-like mechanism

**Figure 2:**
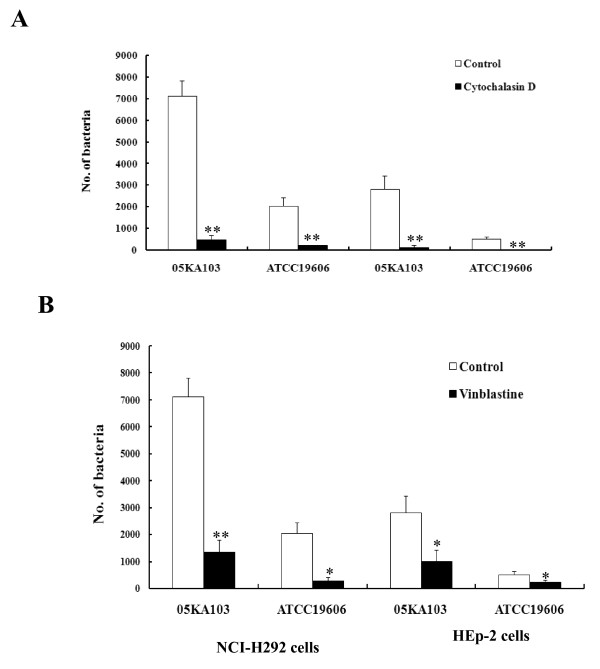
**Inhibition of *A. baumannii *invasion by cytoskeleton inhibitors, cytochalasin D (A) and vinblastine (B)**. NCI-H292 and HEp-2 cells were pretreated with cytochalasin D (2 μM) or vinblastine (100 μM) for 30 min and maintained for 5 h of the entire infection period. *A. baumannii *invasion was measured as the number of bacteria per well using the gentamicin protection assay. Data represent the mean ± standard deviation of three separate experiments. *P < 0.01, **P < 0.005.

To investigate the involvement of microfilaments and microtubules in the cell invasion of *A. baumannii*, epithelial cells were pretreated with cytoskeleton inhibitors, cytochalasin D for microfilaments and vinblastine for microtubules, and a gentamicin protection assay was performed. Both cytochalasin D and vinblastine significantly inhibited the cell invasion of *A. baumannii*. Pretreatment of NCI-H292 cells with cytochalasin D resulted in a decrease of intracellular bacteria of 93% and 90% in *A. baumannii *05KA103 and type strain, respectively (*P *< 0.005) (Fig. 2A). Cell invasion was also significantly decreased by vinblastine (*P *< 0.01) (Fig. 2B). These results suggest that *A. baumannii *invades epithelial cells through both microfilament- and microtubule-dependent uptake mechanisms.

**Figure 3:**
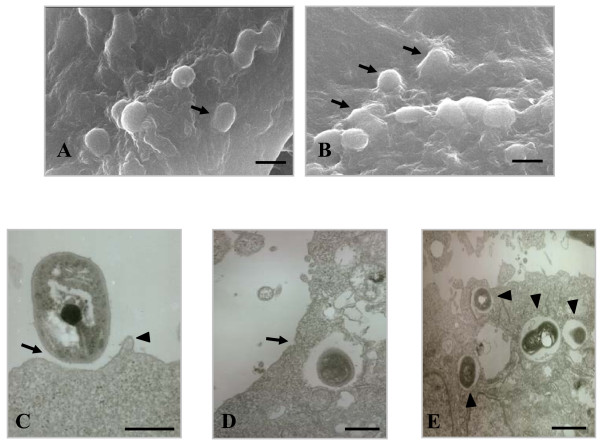
**Electron micrographs demonstrating internalization of *A. baumannii *in epithelial cells**. NCI-H292 cells were infected with *A. baumannii *05KA103 at an MOI of 100 for 5 h. (A and B) SEM images showing interaction of *A. baumannii *with NCI-H292 cells. The cell membrane was extended to and wrapped around *A. baumannii *(arrow). Bar represents 2 μm. (C to E) TEM images. (C) *A. baumannii *loosely anchored to the cell surface (arrow) and small protrusion from the cell was appeared (arrow head). (D) The cell membrane was fused after the bacterial internalization (arrow). (E) Intracellular bacteria were surrounded by a membrane-bound vacuole (arrow head). Bar represents 1 μm.

The association of *A. baumannii *with epithelial cells was characterized in detail by electron microscopy. Scanning electron microscopy (SEM) exhibited the intimate interactions of *A. baumannii *with epithelial cells (Fig. 3A and 3B). The cell membrane extended to and wrapped around bacteria, but the membrane ruffles were not appeared. Transmission electron microscopy (TEM) illuminated the sequential cell invasion of *A. baumannii*. Bacteria were loosely attached to the cell surface and small membrane process protruded from the cells (Fig. 3C). After internalization of *A. baumannii *in epithelial cells, the cell membrane closed at the invasion site (Fig. 3D). Internalized bacteria were located in the membrane-bound vacuoles (Fig. 3E). There was no sign of a membrane ruffle in SEM and TEM images. Cellular changes, such as membrane wrapping around bacteria and fitted vacuoles to bacterial size, suggest that *A. baumannii *is internalized by a zipper-like mechanism, but not a trigger mechanism.

### AbOmpA mediates the interaction of *A. baumannii *with epithelial cells

**Figure 4:**
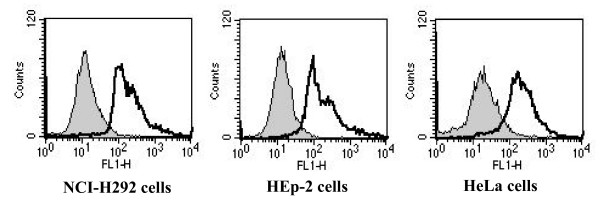
**Cell surface binding of rAbOmpA**. The grey shaded region represents the control fluorescent level seen in the cells treated with polyclonal anti-rabbit AbOmpA antibody and Alexa Fluor ^® ^488-conjugated secondary antibody without the addition of rAbOmpA. The solid line represents the fluorescent level seen in the cells treated with 6 μg/ml of rAbOmpA.

**Figure 5:**
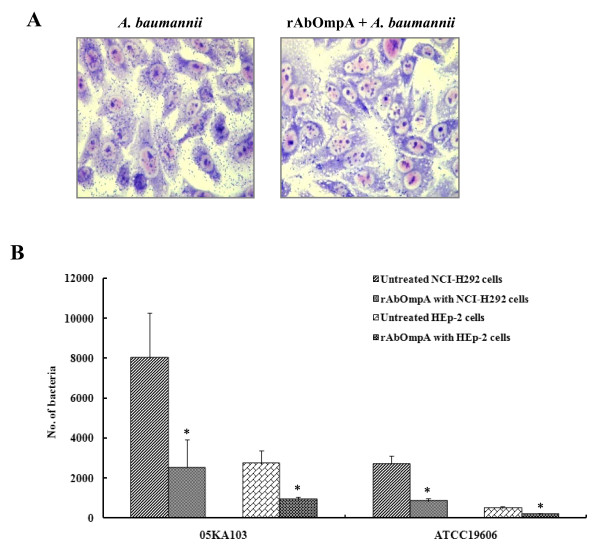
**rAbOmpA attenuates the interaction of *A. baumannii *with epithelial cells**. (A) NCI-H292 cells were pretreated with 10 μg/ml of rAbOmpA for 1 h and then infected with *A. baumannii *05KA103 at an MOI of 100 for 1 h. Cell monolayer was stained with the Giemsa solution. (B) NCI-H292 and HEp-2 cells were pretreated with 10 μg/ml of rAbOmpA for 1 h and infected with *A. baumannii *at an MOI of 100 for 5 h. Invasion efficiency was expressed as the number of bacteria per well using the gentamicin protection assay. The results represent the mean ± standard deviation of three separate experiments. *P < 0.05.

To determine the potential interaction of AbOmpA with epithelial cells, the binding of rAbOmpA to various types of epithelial cells was analyzed by using the flow cytometry. It was found that rAbOmpA specifically bound to the surface of epithelial cells tested (Fig. 4). The mean fluorescence intensities of NCI-H292 and HEp-2 cells were higher than those of HeLa cells, which was in accordance with the cell invasion of *A. baumannii*. We determined whether rAbOmpA inhibited the interactions of *A. baumannii *with epithelial cells. The cytotoxicity of rAbOmpA was first assessed, because ≥ 6 μg/ml of AbOmpA purified from *A. baumannii *ATCC 19606^T ^induced the cytotoxicity of HEp-2 cells [16]. However, ≤ 10 μg/ml of rAbOmpA did not induce any morphological and biochemical changes of epithelial cells for 5 h of incubation (data not shown). NCI-H292 and HEp-2 cells were pretreated with 10 μg/ml of rAbOmpA for 1 h and infected with the highly invasive *A. baumannii *05KA103 for 1 h. As a result, rAbOmpA attenuated the binding of *A. baumannii *to epithelial cells (Fig. 5A). Adherence of *A. baumannii *05KA103 to epithelial cells significantly decreased to 81% in HEp-2 cells (*P *< 0.001) and 87% in NCI-H292 cells (*P *< 0.001). To determine whether rAbOmpA also inhibited the cell invasion of *A. baumannii*, epithelial cells were pretreated with 10 μg/ml of rAbOmpA for 1 h and infected with *A. baumannii *for 5 h. The cell invasion of *A. baumannii *05KA103 significantly decreased to 69% in NCI-H292 cells (*P *< 0.05) and 67% in HEp-2 cells (*P *< 0.05) (Fig. 5B). Collectively, these results suggest that AbOmpA mediates the adherence to and invasion of *A. baumannii *in epithelial cells.

### Cell invasion of isogenic AbOmpA^- ^mutant decreases as compared with wild-type bacteria

**Figure 6:**
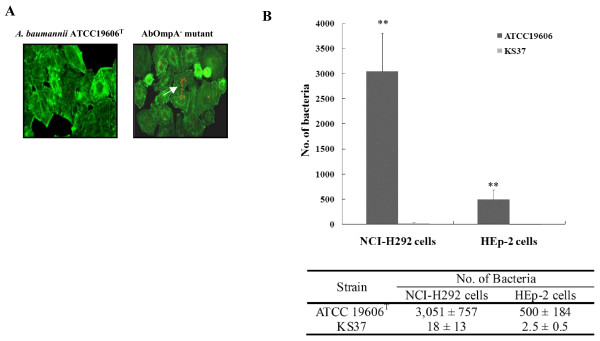
**Adherence and invasion of *A. baumannii *ATCC19606^T ^and isogenic AbOmpA^- ^mutant in epithelial cells**. (A). Adherence of *A. baumannii *to epithelial cells. NCI-H292 cells were infected with *A. baumannii *strains at an MOI of 100 for 1 h. Actin was stained with Alexa Fluor ^® ^488 phalloidin (green). Bacteria were stained with polyclonal anti-rabbit AbOmpA antibody, followed by a secondary antibody Alexa Fluor ^® ^568 (red). (B). NCI-H292 and HEp-2 cells were infected with *A. baumannii *at an MOI of 100 for 5 h. Invasion efficiency was expressed as the number of bacteria per well using the gentamicin protection assay. Results represent the mean and standard deviation of three separate experiments on separately days. *P < 0.001.

To compare the cell adherence of *A. baumannii *ATCC 19606^T ^and its isogenic AbOmpA^- ^mutant KS37, NCI-H292 and HEp-2 cells were infected with bacteria for 1 h. Wild-type bacteria exhibited dispersed adherence (Fig. 6A). The number of adherent bacteria was 1–3 bacteria/infected cells and the percentage of infected cells was less than 3%. The total number of adherent bacteria per 100 cells was 7.5 ± 4.8 in NCI-H292 cells. However, isogenic AbOmpA^- ^mutant formed microcolonies on the surface of epithelial cells, rather than dispersed adherence. The number of adherent bacteria per 100 cells was not significantly different between wild-type bacteria and AbOmpA^- ^mutant. To assess the cell invasion of wild-type bacteria and AbOmpA^- ^mutant, epithelial cells were infected with bacteria for 5 h. The cell invasion of AbOmpA^- ^mutant significantly decreased to 95% as compared with wild-type bacteria (*P *< 0.001) (Fig. 6B). A low cell invasion of AbOmpA^- ^mutant was not due to retarded bacterial growth, because there was no difference in bacterial growth between wild-type bacteria and AbOmpA^- ^mutant (data not shown).

### AbOmpA plays a role in *A. baumannii *pathogenesis *in vivo*

To determine the role of AbOmpA on *A. baumannii *pathogenesis *in vivo*, the murine pneumonia model was introduced. The C57BL/6 mice were intratracheally infected with 1 × 10^9 ^cfu of wild-type bacteria and isogenic AbOmpA^- ^mutant, and euthanized two days after bacterial challenge. Total number of bacterial counts in the lungs were similar between mice infected with wild-type bacteria (1.58 × 10^5 ^cfu/g) and AbOmpA^- ^mutant (6.75 × 10^4 ^cfu/g), but lung histopathology such as the infiltration of polymorphonuclear leukocytes and destruction of alveolar structures was prominently observed in the mice infected with wild-type bacteria (data not shown). Bacteremia occurred in mice infected with both bacterial strains, but there was a significant difference in the bacterial burden between the mice infected with wild-type bacteria (3.5 × 10^3 ^cfu/ml) and isogenic AbOmpA^- ^mutant (2.3 × 10 cfu/ml). These results suggest that AbOmpA is directly associated with the lung pathology and is responsible for the dissemination of *A. baumannii *into the bloodstream.

## Discussion

It was clearly demonstrated that *A. baumannii *has the potential to invade epithelial cells. Host cells play a significant role in determining the cell invasion of *A. baumannii*. Epithelial cells derived from the respiratory tract were more susceptible to *A. baumannii *invasion than epithelial cells derived from non-respiratory tract. Moreover, the binding capacity of rAbOmpA in respiratory tract-derived epithelial cells was higher than that of non-respiratory tract-derived epithelial cells. These findings may be partly responsible for the prevalence of *A. baumannii *in the respiratory tract, which is the most frequent colonization and infection site. The efficiency of cell invasion of *A. baumannii *was lower than other invasive pathogens such as *E. coli, Campylobacter upsaliensis, Helicobacter pylori, N. memingitidis*, *Pseudomonas aeruginosa*, and *Yersinia enterocolitica *[18-21]. A previous study showed that most clinical *A. baumannii *isolates infected only 20–30% of NCI-H292 cells and the number of adhering bacteria was 1–2 bacteria per infected cell [11]. This finding reflects the low cellular invasion of *A. baumannii *in the current study. A relatively low adherence and invasion of *A. baumannii *in epithelial cells may contribute to a low virulence of this opportunistic pathogen, which particularly infects critically ill or severely wounded patients [1,2].

*A. baumannii *invaded epithelial cells by a zipper-like mechanism, as demonstrated by the morphological characteristics. *A. baumannii *was loosely attached to the surface of epithelial cells. This observation is consistent with the previous study regarding adherence of *A. baumannii *to NCI-H292 cells [11]. However, the entrapment of bacteria by cellular protrusions, as noted in the previous study, was not observed. After loose attachment of *A. baumannii *to epithelial cells, the cell membrane was extended to and wrapped around the bacteria, which are typical characteristics of the zipper-like mechanism. These cellular responses against *A. baumannii *were mediated by actin rearrangement and membrane reorganization. Confocal images and gentamicin protection assay with cytochalasin D showed the involvement of actin filaments in the cell invasion of *A. baumannii*. The microtubule inhibitor vinblastine also significantly inhibited the cell invasion of *A. baumannii*. Vinblastine may inhibit the transport of membrane-bound bacteria from the plasma membrane to the cytoplasm by preventing their movement in the microtubule-mediated contractile process. Once inside the host cells, internalized *A. baumannii *was located in the membrane-bound vacuoles. This result, in conjunction with the results of the gentamicin protection assay, suggests that *A. baumannii *survives within membrane-bound vacuoles in the cytoplasm. Many intracellular bacteria are located in a membrane-bound vacuole compartment [22], but some, such as *Neisseria *spp., *Listeria monocytogenes*, *Salmonella *spp., and *Yersinia *spp., escape the cytoplasmic vacuoles and are freely located in the cytoplasm [23-26]. In regards to most pathogenic bacteria, a membrane-bound vacuole containing pathogens does not undergo sequential fusion with endosomes or lysosomes for its own benefit. Further studies are needed to characterize the fate of intracellular *A. baumannii *to understand the survival and dissemination of bacteria.

Outer membrane proteins of Gram-negative bacteria are known to be directly involved in the interaction of bacteria with host cells. The present study focused on whether AbOmpA is responsible for the interaction of *A. baumannii *with epithelial cells. Our data suggest that AbOmpA is a major effector molecule of *A. baumannii *interacting with epithelial cells according to the following experimental results: (i) rAbOmpA directly binds to the surface of epithelial cells; (ii) pre-treatment of rAbOmpA significantly attenuates the binding of *A. baumannii *to epithelial cells; (iii) rAbOmpA significantly inhibits the cell invasion of *A. baumannii*, and (iv) the cell invasion of AbOmpA^- ^mutant decreased to 95% in comparison to wild-type bacteria. In a murine pneumonia model, wild-type bacteria and isogenic AbOmpA^- ^mutant induced different lung pathologies. Moreover, wild-type *A. baumannii *had the capacity to disseminate into the bloodstream, whereas AbOmpA^- ^mutant rarely cross barriers to disseminate in this manner. Accordingly, AbOmpA plays an important role in the *A. baumannii *pathogenesis regarding the induction of pneumonia and bacteremia.

*A. baumannii *can adhere to and invade epithelial cells during colonization and the early stage of infection. In addition to its biological role as a porin, AbOmpA plays a versatile role in the *A. baumannii *pathogenesis regarding its interaction with epithelial cells, the induction of apoptosis of host cells, and the dissemination of bacteria into the bloodstream. Our data provide a novel insight into *A. baumannii *pathogenesis and contribute to further our knowledge of *A. baumannii *infections.

## Conclusion

*A. baumannii *invades epithelial cells depending on both bacterial strains and cell types. Cellular invasion is mediated by both microfilament- and microtubule-dependent uptake mechanisms. AbOmpA is a microbial component responsible for the adherence to and invasion of *A. baumannii *in epithelial cells. The data obtained provide a novel insight into the *A. baumannii *pathogenesis in the early stage of bacterial infection.

## Methods

### Bacterial strains

*A. baumannii *ATCC 19606^T ^and its isogenic AbOmpA^- ^KS37 mutant [16] were used in this study. Four clinical *A. baumannii *isolates were obtained from hospitalized patients: 05KA43 and 05P447 from blood, 05KA103 from cerebrospinal fluid, and 04P275 from wounds. All *A. baumannii *strains were susceptible to gentamicin. The genus *Acinetobacter *was identified by phenotypic markers and species identification was confirmed by amplified ribosomal DNA restriction analysis [27]. For the adherence and invasion assays, *A. baumannii *strains were grown on blood agar plates at 37°C for 18 h and bacterial cells were suspended in a cell culture medium without antibiotics at a density of 1.0 × 10^8 ^cfu/ml.

### Cell cultures

Human laryngeal epithelial cells (HEp-2, CCL-23) and cervical carcinoma cells (HeLa, CCL-2) were cultured in Dulbecco's modified Eagle's medium supplemented with 2 mM L-glutamine, 1000 U of penicillin G per ml, 50 μg of streptomycin per ml, and 10% fetal bovine serum. Human bronchial epithelial cells (NCI-H292, CRL-1848) were cultured in a RPMI 1640 medium containing the above supplements. The cells were maintained at 37°C in 5% CO_2_. Confluent growth was obtained in 100 mm diameter dishes. All cell culture media and supplements were purchased from HyClone Laboratories Inc.

### Cloning and purification of rAbOmpA

The cloning of the *ompA *gene and purification of recombinant AbOmpA (rAbOmpA) were performed as described previously [28]. In brief, the *ompA *gene (1,317 bp) of *A. baumannii *ATCC 19606^T ^was amplified by PCR using the upstream primer (5'-ACAGGATCCATGAAATTGAGTCGTATT-3') and downstream primer (5'-ACAAGCTTTTATTGAGCTGCTGCA-3'). PCR products were ligated into the pET28a expression vector (Novagen). *E. coli *BL21 (DE3)/pET28a carrying the *ompA *gene were grown in Luria-Bertani (LB) broth at 37°C and rAbOmpA was overexpressed with 1 mM IPTG at 25°C for 4 h. After bacterial sonication, the supernatant containing the soluble form of rAbOmpA was collected and loaded on a 5 ml HiTrap™ FF column (Amersham Biosciences). His-tagged rAbOmpA was eluted by elution buffer (20 mM sodium phosphate, 500 mM NaCl, and 500 mM imidazole, pH 7.4). The rAbOmpA samples were dialyzed against the elution buffer without imidazole and phosphate-buffered saline (PBS). The rAbOmpA samples were mixed with polymyxin B-agarose (St. Louis. Mo, USA) to remove endotoxin complex. The samples were concentrated by Centricon (2,000 MW cut off; Millipore) and stored at -70°C.

### Antibodies

Polyclonal anti-AbOmpA antiserum against the purified OmpA from *A. baumannii *ATCC 19606^T ^was raised in rabbits by routine immunogenic procedures [29].

### Bacterial adherence assay

In each well of a 24 well cell culture plates, *A. baumannii *was added to a monolayer of epithelial cells at a ratio of bacteria to host cells of 100:1 (MOI of 100). The cells infected with bacteria were incubated in a 5% CO_2 _at 37°C for 1 h. The cells were washed five times with PBS, fixed with methanol for 20 min, and stained with Giemsa solution [11].

### Bacterial invasion assay

Epithelial cells grown in 24 well plates were infected with *A. baumannii *for the indicated times at an MOI of 100. Culture media were removed and the cell monolayer was washed three times with PBS. A fresh culture medium containing 300 μg/ml of gentamicin was added and incubated for another 2 h. The cells were washed five times with PBS and lysed with 0.1% Triton X-100 at 37°C for 20 min. Dilutions from each well were plated on nutrient agar and colonies were enumerated 20 h after incubation. The invasion efficiency was calculated as the average number of bacteria per well. Each invasion assay was performed in duplicate and repeated on a minimum of three separate days. To determine the effect of cytoskeleton inhibitors on *A. baumannii *invasion, 2 μM of cytochalasin D (Sigma) or 100 μM of vinblastine (Sigma) was added to the cells 30 min before the addition of bacteria and maintained in the medium for the entire infection period. Neither cytoskeletal inhibitors affected the bacterial viability and cellular viability at the concentrations used. To determine the inhibitory effect of AbOmpA on bacterial invasion, epithelial cells were pretreated with 10 μg/ml of rAbOmpA 1 h before bacteria infection.

### Cell surface binding of rAbOmpA

Epithelial cells were grown in 100 mm dishes and then detached from the dishes by treatment with 0.5 M EDTA solution. The cells were washed three times and suspended at 5 × 10^5 ^cells/ml in PBS. The cells were incubated in the presence of polyclonal anti-rabbit AbOmpA antibody (1:1,000). After washing, the cells were incubated with 6 μg/ml of rAbOmpA at 4°C for 20 min and washed three times in cold PBS. The samples were then incubated with anti-rabbit AbOmpA antibody (1:1,000) on ice for 1 h. The cells were washed three times with cold PBS and incubated with Alexa Fluor^®^488-conjugated anti-rabbit IgG (1:500) on ice for 1 h. The labelled cells were analyzed by flow cytometry (Becton Dickinson).

### Fluorescence and confocal microscopy

NCI-H292 cells were grown on 13 mm diameter glass coverslips at 2 × 10^5 ^per coverslip. *A. baumannii *was infected at an MOI of 100 and incubated at 37°C for 5 h. The coverslips were washed with PBS and fixed with 3.7% paraformaldehyde for 30 min. Cells were permeabilized with a PBS containing 0.25% Triton X-100 for 10 min. Actin was stained with Alexa Fluor^®^488 phalloidin (Molecular Probes). *A. baumannii *was labeled with polyclonal anti-rabbit AbOmpA antibody (1:1,000), followed by Alexa Fluor^®^568-conjugated goat anti-rabbit IgG antibody (Molecular Probes). The association of *A. baumannii *with epithelial cells was observed either with a Nikon inverted fluorescence microscope or Leica confocal microscope.

### TEM and SEM

For TEM, NCI-H292 cells were grown in 35 mm culture dishes at a concentration of 1 × 10^6 ^cells/well. *A. baumannii *was infected to the monolayer of NCI-H292 cells at an MOI of 100 for 5 h. The cells were washed five times with PBS and harvested by using trypsin-EDTA (500 mg/ml trypsin, 200 mg/ml EDTA). The cells were centrifuged at 300 g for 5 min and washed with PBS. The cells were resuspended in a fixative (4% paraformaldehyde and 1% glutaraldehyde, pH 7.0). The samples were then incubated in osmium tetroxide, gradually dehydrated in a series of ethanol and embedded in Epon. Thin sections were prepared by using an ultramicrotome with a diamond knife. The samples were examined by a Hitachi 7000 transmission electron microscope. For SEM, NCI-H292 cells were grown on 13 mm diameter glass coverslips at a concentration of 2 × 10^5 ^cells/well and bacteria were infected at an MOI of 100 for 5 h. The samples were washed with PBS and fixed with 2.5% glutaraldehyde at room temperature. The cells were gradually dehydrated in a series of ethanol and dried to a critical point. The samples were then coated with a layer of gold and examined under 15 kV accelerating voltage in a Hitachi S-4300 field emission scanning electron microscope.

### Animal experiments

Six-week-old female C57BL/6 mice were maintained under specific-pathogen-free conditions. For the pneumonia model, the mice were anesthetized with pentobarbital and then 100 μl of 1 × 10^10 ^cfu/ml of bacteria were administrated intratracheally. Groups containing three mice were infected with wild-type *A. baumannii *and isogenic AbOmpA^- ^mutant. The control mice groups were injected with 100 μl of PBS. The animals were suspended in a supine position on a 60° incline board. Their trachea was exposed surgically and bacteria were introduced intratracheally via a syringe with a 30 1/2 gauged-needle. The incisions were closed by surgical sutures. The bacterial density of each run was confirmed by serial dilution and culture of an aliquot from each inoculum. The lungs of mice infected with bacteria were removed two days after bacterial challenge. For histological analysis, tissues were stained with hematoxylin/eosin and observed by using a Nikon microscope. For bacterial counts, lungs were homogenized by using a glass syringe piston and then serial 10-fold suspension was inoculated onto MacConkey agar plates (Difco Laboratories). Blood was directly plated on a MacConkey agar plate.

### Statistical analysis

The statistical significance of the data was determined by the Student's *t*-test. A *P *value of < 0.05 was considered to be statistically significant.

## Abbreviations

AbOmpA: Outer membrane protein A of *A. baumannii*; PBS: phosphate-buffered saline; LB broth: Luria-Bertani broth.

## Authors' contributions

CH designed this study, carried out all experimental work, and completed the data analysis. JS performed the FACS experiments. YC participated in the data analysis and evaluation of the results. TI contributed to the electron microscopy experiments. JC coordinated the study, assisted in writing the manuscript, and revised the final manuscript. All authors read and approved the manuscript.

## References

[B1] Dijkshoorn L, Nemec A, Seifert H (2007). An increasing threat in hospitals: multidrug-resistant *Acinetobacter baumannii*. Nat Rev Microbiol.

[B2] Peleg AY, Seifert H, Paterson DL (2008). *Acinetobacter baumannii*: emergence of a successful pathogen. Clin Microbiol Rev.

[B3] Go ES, Urban C, Burns J, Kreiswirth B, Eisner W, Mariano N, Mosinka-Snipas K, Rahal JJ (1994). Clinical and molecular epidemiology of *Acinetobacter *infections sensitive only to polymyxin B and sulbactam. Lancet.

[B4] Seifert H, Strate A, Schulze A, Pulverer G (1994). Bacteremia due to *Acinetobacter *species other than *Acinetobacter baumannii*. Infection.

[B5] Nemec A, Dijkshoorn L, Cleenwerck I, De Baere T, Janssens D, Reijden TJ van der, Jezek P, Vaneechoutte M (2003). *Acinetobacter parvus *sp. Nov., a small-colony-forming species isolated from human clinical specimens. Int J Sys Evol Microbiol.

[B6] Fournier PE, Richet H (2006). The epidemiology and control of *Acinetobacter baumannii *in health care facilities. Clin Infect Dis.

[B7] Naiemi NA, Duim B, Savelkoul PH, Spanjaard L, de Jonge E, Bart A, Vandenbroucke-Grauls CM, de Jong MD (2005). Widespread transfer of resistance genes between bacterial species in an intensive care unit: implications for hospital epidemiology. J Clin Microbiol.

[B8] Beachey EH (1981). Adhesin-receptor interactions mediating the attachment of bacteria to mucosal surfaces. J Infect Dis.

[B9] Alonso A, García-del Portillo F (2004). Hijacking of eukaryotic functions by intracellular bacterial pathogens. Int Microbiol.

[B10] Dramsi S, Cossart P (1998). Intracellular pathogens and the actin cytoskeleton. Annu Rev Cell Dev Biol.

[B11] Lee JC, Koerten H, Broek P van den, Beekhuizen H, Wolterbeek R, Barselaar M van den, Reijden T Van der, Meer J Van der, Gevel J Van de, Dijkshoorn L (2006). Adherence of *Acinetobacter baumannii *strains to human bronchial epithelial cells. Res Microbiol.

[B12] Khan NA, Shin S, Chung JW, Kim KJ, Elliott S, Wang Y, Kim KS (2003). Outer membrane protein A and cytotoxic necrotizing factor-1 use diverse signaling mechanisms for *Escherichia coli *K1 invasion of human brain microvascular endothelial cells. Microb Pathog.

[B13] Moore J, Bailey SE, Benmechernene Z, Tzitzilonis C, Griffiths NJ, Virji M, Derrick JP (2005). Recognition of saccharides by the OpcA, OpaD, and OpaB outer membrane proteins from *Neisseria meningitides*. J Biol Chem.

[B14] Pal U, Yang X, Chen M, Bockenstedt LK, Anderson JF, Flavell RA, Norgard MV, Fikrig E (2004). OspC facilitates *Borrelia burgdorferi *invasion of *Ixodes scapularis *salivary glands. J Clin Invest.

[B15] Prasadarao NV, Wass CA, Weiser JN, Stins MF, Huang SH, Kim KS (1996). Outer membrane protein A of *Escherichia coli *contributes to invasion of brain microvascular endothelial cells. Infect Immun.

[B16] Choi CH, Lee EY, Lee YC, Park TI, Kim HJ, Hyun SH, Kim SA, Lee SK, Lee JC (2005). Outer membrane protein 38 of *Acinetobacter baumannii *localizes to the mitochondria and induces apoptosis of epithelial cells. Cell Microbiol.

[B17] Choi CH, Hyun SH, Lee JY, Lee JS, Lee YS, Kim SA, Chae JP, Yoo SM, Lee JC (2008). *Acinetobacter baumannii *outer membrane protein A targets the nucleus and induces cytotoxicity. Cell Microbiol.

[B18] Capecchi B, Adu-Bobie J, Di Marcello F, Ciucchi L, Masignani V, Taddei A, Rappuoli R, Pizza M, Arico B (2005). *Neisseria meningitidis *NadA is a new invasin which promotes bacterial adhesion to and penetration into human epithelial cells. Mol Microbiol.

[B19] Fleiszig SM, Zaidi TS, Pier GB (1995). *Pseudomonas aeruginosa *invasion of and multiplication within corneal epithelial cells *in vitro*. Infect Immun.

[B20] Kwok T, Backert S, Schwarz S, Berger J, Meyer TF (2002). Specific entry of *Helicobacter pylori *into cultured gastric epithelial cells via a zipper-like mechanism. Infect Immun.

[B21] Mooney A, Byrne C, Clyne M, Johnson-Henry K, Sherman P, Bourke B (2003). Invasion of human epithelial cells by *Campylobacter upsaliensis*. Cell Microbiol.

[B22] Sinai AP, Joiner KA (1997). Safe haven: the cell biology of nonfusogenic pathogen vacuoles. Annu Rev Microbiol.

[B23] Isberg RR (1991). Discrimination between intracellular uptake and surface adhesion of bacterial pathogens. Science.

[B24] Jarvis GA, Li J, Swanson KV (1999). Invasion of human mucosal epithelial cells by *Neisseria gonorrhoeae *upregulates expression of intercellular adhesion molecule 1 (ICAM-1). Infect Immun.

[B25] Moulder JW (1985). Comparative biology of intracellular parasitism. Microbiol Rev.

[B26] Tang P, Rosenshine I, Finlay BB (1994). *Listeria monocytogenes*, an invasive bacterium, stimulates MAP kinase upon attachment to epithelial cells. Mol Biol Cell.

[B27] Vaneechoutte M, Dijkshoorn L, Tjernberg I, Elaichouni A, de Vos P, Claeys G, Verschraegen G (1995). Identification of *Acinetobacter *genomic species by amplified ribosomal DNA restriction analysis. J Clin Microbiol.

[B28] Lee JS, Lee JC, Lee CM, Jung ID, Jeong YI, Seong EY, Chung HY, Park YM (2007). Outer membrane protein A of *Acinetobacter baumannii *induces differentiation of CD4^+ ^T cells towards a Th1 polarization phenotype through the activation of dendritic cells. Biochem Pharmacol.

[B29] Hanly WC, Artwohl JE, Bennett BT (1995). Review of polyclonal antibody production procedures in mammals and poultry. ILAR J.

